# MicroRNA-96 Directly Inhibits γ-Globin Expression in Human Erythropoiesis

**DOI:** 10.1371/journal.pone.0022838

**Published:** 2011-07-28

**Authors:** Imane Azzouzi, Hansjoerg Moest, Jeannine Winkler, Jean-Claude Fauchère, André P. Gerber, Bernd Wollscheid, Markus Stoffel, Markus Schmugge, Oliver Speer

**Affiliations:** 1 Division of Haematology, University Children's Hospital, Zurich, Switzerland; 2 Research Center for Children, University Children's Hospital, Zurich, Switzerland; 3 Zurich Center for Integrative Human Physiology, University of Zurich, Zurich, Switzerland; 4 Institute of Molecular Systems Biology, ETH Zurich, Zurich, Switzerland; 5 Division of Neonatology, University Hospital Zurich, Zurich, Switzerland; 6 Institute of Pharmaceutical Sciences, ETH Zurich, Zurich, Switzerland; French National Center for Scientific Research - Institut de biologie moléculaire et cellulaire, France

## Abstract

Fetal hemoglobin, HbF (α_2_γ_2_), is the main hemoglobin synthesized up to birth, but it subsequently declines and adult hemoglobin, HbA (α_2_β_2_), becomes predominant. Several studies have indicated that expression of the HbF subunit γ-globin might be regulated post-transcriptionally. This could be confered by ∼22-nucleotide long microRNAs that associate with argonaute proteins to specifically target γ-globin mRNAs and inhibit protein expression. Indeed, applying immunopurifications, we found that γ-globin mRNA was associated with argonaute 2 isolated from reticulocytes that contain low levels of HbF (<1%), whereas association was significantly lower in reticulocytes with high levels of HbF (90%). Comparing microRNA expression in reticulocytes from cord blood and adult blood, we identified several miRNAs that were preferentially expressed in adults, among them miRNA-96. The overexpression of microRNA-96 in human *ex vivo* erythropoiesis decreased γ-globin expression by 50%, whereas the knock-down of endogenous microRNA-96 increased γ-globin expression by 20%. Moreover, luciferase reporter assays showed that microRNA-96 negatively regulates expression of γ-globin in HEK293 cells, which depends on a seedless but highly complementary target site located within the coding sequence of γ-globin. Based on these results we conclude that microRNA-96 directly suppresses γ-globin expression and thus contributes to HbF regulation.

## Introduction

The major hemoglobin in the fetus is hemoglobin F (HbF; α_2_γ_2_), whereas in adult humans mainly hemoglobin A (HbA; α_2_β_2_), and, to a lesser extent, hemoglobin A_2_ (α_2_δ_2_) are expressed [1], [2]. In many hemoglobinopathies HbF expression persists or can be induced by drugs, and it is known that increased HbF expression is beneficial, as it can compensate for reduced or abnormal HbA expression [3], [4]. In an effort to understand the molecular regulation of HbF expression numerous studies have identified cis-acting DNA elements flanking the γ-globin gene, and several transcription factors that bind to these elements have been characterized [1], [5]. They form chromatin-protein complexes activating the γ-globin transcription in fetal-embryonic erythropoiesis, favoring HbF expression. After birth these complexes are remodeled, silencing the γ-globin loci and activating the β-globin loci, and hemoglobin expression is switched to HbA [1], [2]. Besides such transcriptional control, several studies have indicated that hemoglobin expression may also be post-transcriptionally regulated [6], [7], [8]. For instance, reticulocytes (immature erythrocytes) isolated from sickle cell disease patients after treatment with butyrate showed a significant increase in HbF protein, but no changes in γ-globin mRNA levels [7]. Moreover, γ-globin transcription rates [8] or γ-globin mRNA levels [6] were higher than expected from the low HbF protein levels measured in patients with Corfu δβ-thalassemia and β-thalassemia, respectively. Although these findings indicate that HbF expression in reticulocytes may be post-transcriptionally regulated, there is no formal proof nor are the underlying molecular mechanisms known to date.

MicroRNAs (miRNAs or miRs) are small, 19 to 25 nucleotide long, non-coding RNAs, which target mRNAs in a sequence-specific manner, inducing translational repression or decay [9], [10]. Following nuclear processing, miRNA precursors (pre-miRNAs) are exported to the cytoplasm and converted into mature miRNAs by Dicer [11]; one-strand of the duplex is subsequently incorporated into miRNA-induced silencing complexes (miRISC) comprised of a member of the argonaute (AGO) protein family and importin 8 (Imp8) [12]. This complex assembles with sequences located mostly in the 3′-UTRs of target mRNAs. Although the rules of miRNA-target recognition are not yet fully established, one determinant is the complementarity between the target site and 6–7 nucleotides at the 5′ end of the miRNA (region known as miRNA “seed” and reviewed in [11]).

In humans, miRNAs have been detected in granulocytes, monocytes, lymphocytes, platelets [13], [14], during erythropoiesis [15], and in red blood cells (RBCs) [16], [17]. In both types of enucleated cells (platelets and RBCs), miRNAs have been shown to regulate cell-type specific proteins [14], [16]. More than 200 miRNAs have been identified in RBCs [16], [18]. In reticulocytes, miR-320 was shown to regulate the expression of the transferrin receptor CD71 [16]. Additionally, elevated miR-210 levels have been studied in the context of elevated γ-globin levels in two cases of hereditary persistence of HbF [19], while the let-7 family has been associated with hemoglobin switching [18]. Recently, two miRNAs, miR-221 and miR-222, have been identified to regulate HbF expression in erythropoietic cells via regulation of the kit receptor [20]. Further indirect regulation of HbF expression by miRNAs was shown in trisomy 13 cases, in which miR-15 and miR-16 levels were elevated, resulting in an enhanced down-regulation of MYB, an inhibitor of the γ-globin gene transcription [21]. Nevertheless, whether γ-globin mRNAs could also be directly targeted by certain miRNAs has not been reported yet.

First, we demonstrate that γ-globin mRNA is bound by AGO2-containing miRISC in reticulocytes from adults with 0.5% HbF, but less so in reticulocytes from umbilical cord blood with 90% HbF. Secondly, we report that miRNA-96, miRNA-146a, let-7a, miR-888 and miR-330a-3p are significantly more abundant in reticulocytes obtained from adults than from umbilical cord blood. Thirdly, we show that ectopic expression of miR-96 during *ex-vivo* erythropoiesis suppresses HbF expression, whereas knockdown of miR-96 increases HbF expression. Finally, we demonstrate that miRNA-96 directly targets the ORF of γ-globin mRNA. These findings demonstrate that miRNAs contribute to HbF regulation by the post-transcriptional inhibition of γ-globin expression during adult erythropoiesis.

## Results

### γ-globin mRNA is bound by AGO2

Reticulocytes (immature erythrocytes) synthesize up to 20% of their hemoglobin content after extrusion of the nucleus and release from the bone marrow [22]. Several studies have indicated that HbF expression in reticulocytes might be regulated post-transcriptionally [6], [7], [23]. Although miRNA expression profiling showed a number of miRNAs to be present in reticulocytes [16], [18], to our knowledge, no study has reported whether miRNAs regulate HbF expression during erythropoiesis or in reticulocytes. In order to clarify whether globin mRNAs might be targeted by miRNAs respectively bound my miRISC, we decided to compare in a first step the miRISC composition in reticulocytes from umbilical cord blood (CB) expressing high HbF (90.3±1.0%) with reticulocytes from healthy adult blood (AB) expressing low HbF (0.9±0.3%) levels.

**Figure 1:**
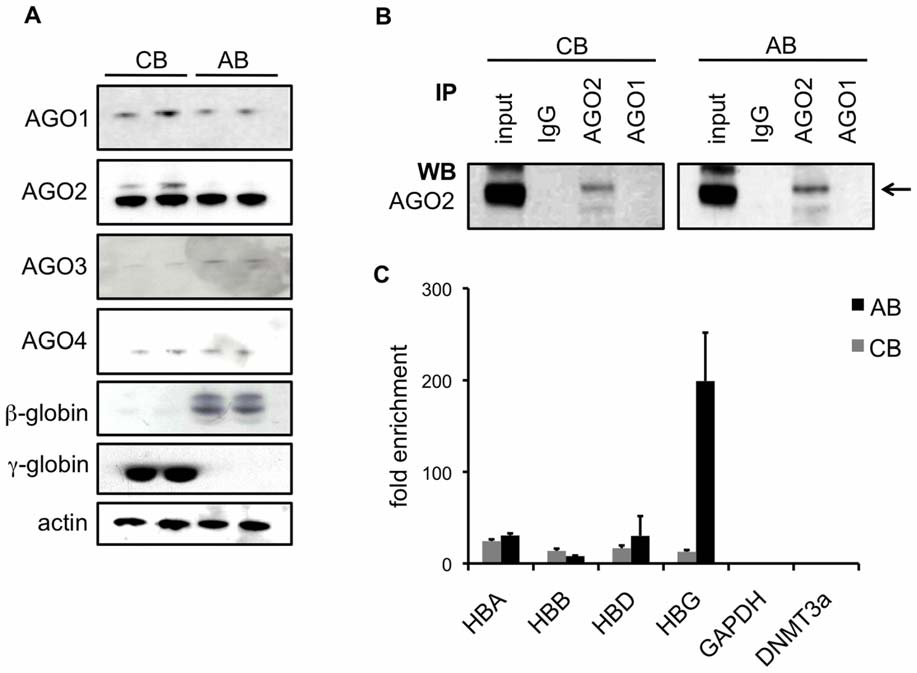
γ-globin mRNA is bound by AGO2 in reticulocytes with low HbF content. (A) Western blot analysis of AGO1, 2, 3, 4, γ-globin and β-globin in reticulocytes purified from umbilical cord blood (*CB*) and blood from adults (*AB*). Actin was included as loading control. (B) AGO2-containing RNA-protein complexes were immunoprecipitated from equal amounts of cord blood reticulocyte (*CB*) and adult blood reticulocyte (*AB*) lysates using rat monoclonal anti-AGO2 antibodies; as isotype control, non-specific rat immuno-globulin (*IgG*) as well as rat monoclonal anti-AGO1 antibodies were used. Immunoprecipitations were compared to lysate (*input*) and subjected to immunoblot analysis using anti-AGO2 antibodies. (C) The amounts of α-globin (*HBA*), β-globin (*HBB*), γ-globin (*HBG*) and δ-globin (*HBD*) mRNA immunoprecipitated together with AGO2 from CB and AB were compared to amounts of globin mRNAs that were non-specifically immunoprecipitated with control IgG. As control GAPDH and DNMT3a mRNAs were analyzed. For every sample the same amount of precipitated RNA was analyzed. The values are expressed as fold-enrichment over IgG immunoprecipitations, and represent mean±SEM (n = 3).

After probing with specific anti-human AGO antibodies, we detected all 4 human AGO proteins AGO1, AGO2, AGO3 and AGO4 proteins in reticulocytes from AB and CB (Figure 1A). Next we decided to study AGO2 in further detail by investigating whether AGO2 is associated with globin mRNAs. Accordingly, we performed immunoprecipitations (IP) of AGO2 from AB and CB reticulocytes (Figure 1B) using previously characterized monoclonal rat anti-AGO2 antibodies [24], [25], [26]. Within these immunoprecipitated miRISCs, we subsequently compared the amount of AGO2-bound α-, β-, γ- and δ-globin mRNAs by quantitative real time PCR (qPCR). From AB reticulocyte lysates, α-globin mRNA was enriched 30.6±2.3 fold, β-globin 7.9±0.7 fold, δ-globin 30.1±21.6 fold and γ-globin 199.2±52.5 fold (mean±SEM) in AGO2 IP compared to control IP performed with non-specific rat IgG. Strikingly, from CB reticulocyte lysates, γ-globin mRNA was enriched 12.2±0.7 fold, i.e.15 times less than from AB reticulocytes, whereas β-globin was enriched 10.8±1 fold, almost two times more than from AB reticulocytes (Figure 1C). In contrast, after quantifying γ-globin mRNA molecules we found that reticulocytes from CB contain on average 393±19 copies per cell, whereas reticulocytes from AB contain 16±12 copies per cell, indicating that the majority of γ-globin mRNAs are bound by AGO2 in AB. Two control transcripts, glyceraldehyde 3-phosphate dehydrogenase (GAPDH) and DNA methyltransferase 3a (DNMT3a), could not be detected in AGO2 IP from AB and CB reticulocytes (Figure 1C), demonstrating the specificity of the IP. In addition, no globin mRNAs could be detected in AGO1 IPs (Figure 1B; data not shown). Unfortunately anti-AGO3 and anti-AGO4 antibodies did not precipitate AGO proteins at detectable levels, within these precipitations neither globin mRNAs were detected.

In order to verify the presence of AGO2 in the immunoprecipitated miRISC, IP samples were analyzed by liquid chromatography-coupled tandem mass spectrometry (LC-MS/MS). 12 unique peptides for AGO2 were detected in AGO2 IP samples (Figure S2), whereas no AGO peptides were found in the IgG control samples.

### Distinct miRNA patterns predict miRNAs targeting γ-globin mRNA

**Figure 2:**
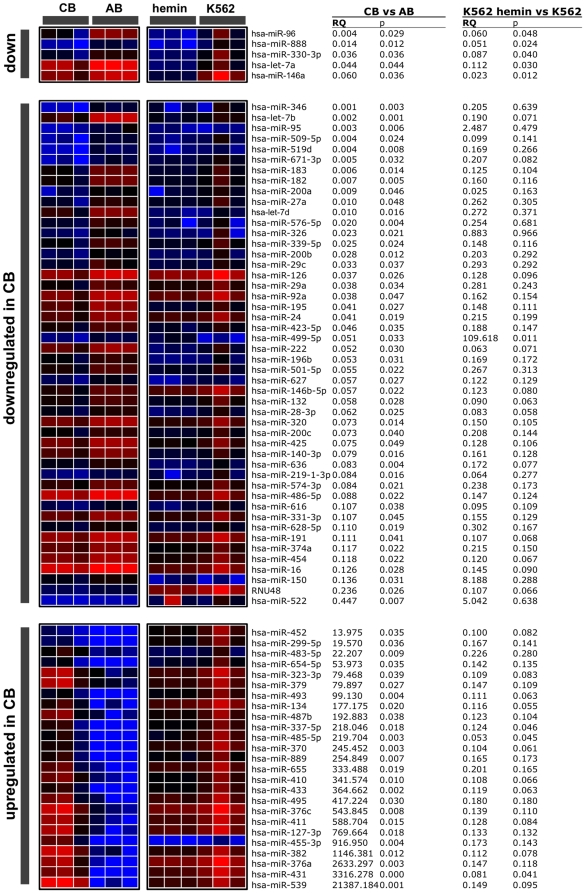
miRNA expression-patterns in cells expressing high HbF-levels differ from cells with low HbF-levels. Total RNA isolated from reticulocytes from cord blood (*CB*) (n = 3), adult blood (*AB*) (n = 3), K562 cells treated with hemin (n = 3) and untreated K562 cells (n = 3) was used to generate miRNA expression profiles. Red color indicates higher expression and blue color lower expression compared to the global mean. miRNAs were classified into three groups: miRNAs significantly less expressed in cells with high HbF content, i.e. CB compared to AB and K562 cells treated with hemin compared to untreated K562 cells (*down*), miRNAs significantly less expressed in CB compared to AB (*downregulated in CB*), miRNAs less expressed in cells with high HbF content (*upregulated in CB*). The relative quantification (*RQ*) values, representing the fold enrichment, and the corresponding p values (determined by two tailed Student's t-test) are presented.

**Figure 3:**
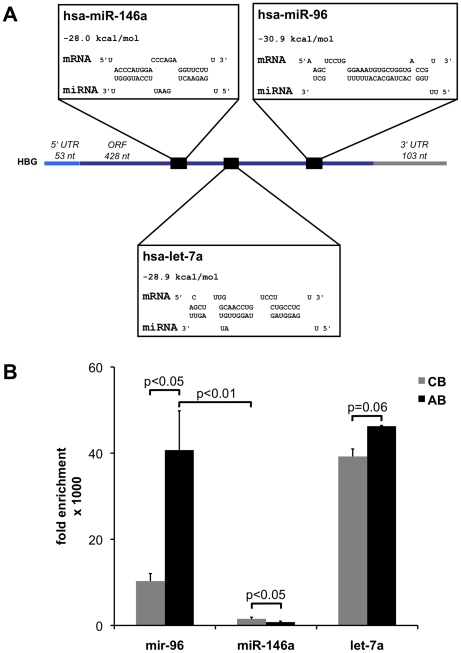
miR-96, miR-146a, let-7a are predicted to target the open reading frame of the γ-globin mRNA. (A) Represented are the 5′ untranslated region (5′UTR, light blue), open reading frame (ORF, dark blue) and 3′UTR (gray) of γ-globin mRNA (HBG). By using RNA hybrid [32], the free energy of the individual miRNA:mRNA hybridization was determined and the corresponding base pairing shown. The hybridizations of γ-globin mRNA with miR-96, miR-146a and let-7a (black squares) showed either well-defined mRNA:miRNA pairing (miR-146a and let-7a) consisting of a seed region containing eight base pairs, followed by a four-base pair bulge region and a 3′ complementary region as described [11] or seedless base pairing (miR-96), all within the open reading frame (ORF) of HBG. (B) The amounts of miR-96, miR-146a and let-7a immunoprecipitated together with AGO2 from CB and AB were compared to amounts of miR-96, miR-146a and let-7a that were non-specifically immunoprecipitated with control IgG. For every sample the same amount of precipitated RNA was analyzed. The values are expressed as 1000-fold-enrichment over IgG immunoprecipitations, and represent mean±SEM (n = 3). P values were determined by the Student's t-test.

To gain insight into which miRNAs might contribute to the regulation of HbF expression in reticulocytes, we analyzed miRNA profiles in CB and AB reticulocytes. In both groups of reticulocytes 221 different miRNAs were could be quantified by qPCR (Table S1). Among them, 190 miRNAs were less expressed in CB compared to AB reticulocytes. To reduce this list of 190 miRNAs, we employed an artificial *in vitro* system: we isolated a clone of the myelogenous leukemia cell line K562 that did not express HbF at detectable levels, whereas HbF increased to almost 50% of the expressed hemoglobin after treatment with hemin [27], [28], [29]. However, the Philadelphia chromosome (Ph1)-positive K562 cells cannot be compared to any normal stage of erythropoiesis, neither before nor after hemin treatment [30]. Therefore we did not regard this K562 clone as a model for erythropoiesis or for a shift from CB to AB. Nonetheless, we compared miRNA expression patterns of K562 cells before and after hemin-induced HbF synthesis. Only five miRNAs - miR-96, miR-888, miR330-3p, let-7a, and miR-146a - were significantly less abundant in CB reticulocytes compared to AB reticulocytes and were also significantly down-regulated in hemin treated K562 cells (Figure 2). None of these five miRNAs was predicted to target γ-globin mRNA by TargetScan [31], a database representing computationally predicted mRNA targets for miRNAs. However, RNAhybrid [32] predicted that miR-96, miR-146a and let-7a could target γ-globin mRNA (Figure 3A). The program predicted a seedless but highly complementary hybridization with few mismatches for miR-96, whereas it predicted canonical target hybridization with a seed sequence, a central loop formation and a 3′ complementary region for miR-146a and let-7a. Interestingly, these miRNAs were predicted to hybridize to target sequences located within the open reading frame (ORF) of the γ-globin mRNA (Figure 3A) but not within other globin mRNAs (data not shown). Consequently we compared the amount of miR-96, miR-146a and let-7a bound to AGO2 similarly as done for the globin mRNAs. Surprisingly only miR-96 enriched significantly more in AGO2 complexes immunoprecipitated from AB compared to CB. In contrast miR-146 and let-7a showed no significant difference in enrichment in AB compared to CB (Figure 3B). This result was the first indication that probably miR-96 but not miR-146 and let-7a might directly target the γ-globin mRNA.

### miR-96 inhibits γ-globin expression in human erythropoiesis

**Figure 4:**
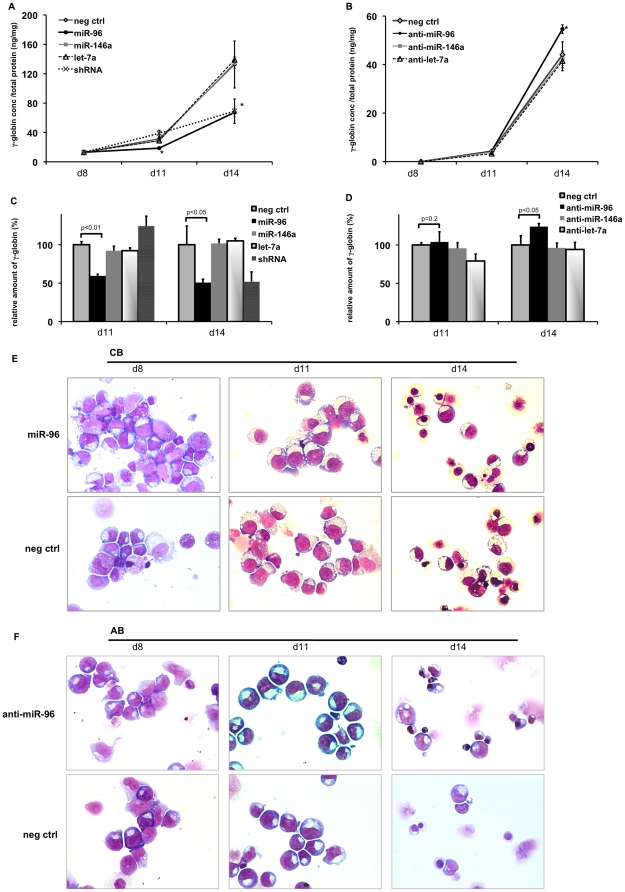
miR-96 inhibits γ-globin expression in human erythropoiesis. (A,C,E) Cord blood-derived erythroid cultures (CB) were transduced with miRNA precursors; (B,D,F) Adult bone marrow-derived erythroid cultures (BM) were transduced with anti-miRNAs. Cells were harvested and analyzed at day 8 (*d8*), 11 (*d11*) and 14 (*d14*). (A–B) Specific amounts of γ-globin per total protein concentration and (C–D) relative amounts of γ-globin compared to negative control, which was set to 100%, were measured by ELISA. Values represent mean ± SEM of 3 experiments (n = 3). P values were determined by the Student's t-test. * p<0.05. (E–F) Representative cytospins of erythroid cells on *d8*, *d11* and *d14*. The cytospins were stained with May Grünwald Giemsa and images were acquired with a Zeiss Axioskop2 microscope equipped with a Zeiss Plan-Apochromat 63×/1.4 oil immersion objective lens and a Zeiss AxioCam MRc digital camera. Images were recorded using Zeiss AxioVision AC release 4.5.0 software.

To investigate whether increased expression of miR-96, miR-146a or let-7a in CB derived erythropoiesis leads to lower γ-globin expression, CB derived erythroblasts were transduced with vectors encoding miRNA precursors for miR-96, miR-146a, let-7a, shRNA against γ-globin, and as a control, with vectors without any insert. At day 11 and 14 in culture, levels of miR-96, miR-146a, let-7a increased more than 20 times over the endogenous level (Figure S3 A), reaching 10 fold higher miR-96 levels compared to the respective stage of adult erythropoietic cells. Non-transduced erythropoietic cells as well as cells transduced with empty vector started to express γ-globin after erythropoietin (EPO) stimulation. At day 11 (d11) the γ-globin was doubled, which further increased more than 10 fold at d14. Erythropoietic cells over-expressing miR-96 had about half of the γ-globin levels of non-treated cells. This was comparable to levels when γ-globin is specifically knocked-down with shRNA. On the other hand, the over-expression of miR-146a and let-7a did not influence γ-globin expression levels (Figure 4 A, C).

To complement our set of experiments, after EPO stimulation bone marrow (BM) derived erythroblasts were transduced with vectors expressing anti-miRNAs. Only at day 14 of the culture, the endogenous levels of miR-96, miR-146 and let-7a were decreased to almost 50% of the levels seen in negative control cells, transduced with scambled-RNAs (Figure S3 B). At this time point, erythropoietic cells transduced with anti-miR-96 significantly increased their γ-globin content by 20% compared to the negative control, but also compared to cells transduced with anti-miR -146a and anti-let -7a (Figure 4 B, D). In addition we also incubated BM-derived erythroblasts with antagomirs [33], which led to approximately 90% depletion of miR-96, miR-146 and let-7a; however effects on γ-globin expression were similar compared to viral encoded anti-miRs (data not shown).

Important to note: neither miR-96 over expression nor miR-96 knock down led to a change of β-globin as shown by Western blot (Figure S3E), which was expected, as none of the investigated miRNAs was predicted to have a target site within the β-globin mRNA.

After quantifying developmental stages during in vitro erythropoiesis, we observed that let-7a seemed to influence differentiation: after overexpression miR-146 and let-7a in CB-derived erythropoiesis the relative abundance of late erythroblasts was significantly decreased, whereas the relative abundance of early erythroblasts and intermediate erythroblasts were increased. Consistently after knockdown of let-7a in BM-derived erythropoiesis the relative abundance of early erythroblasts significantly decreased, whereas intermediate and late erythroblasts significantly increased. The same trend was observed for miR-146a but was not significant. However neither miR-96 overexpression nor knockdown showed any change in the differentiation stages of erythropoiesis (Table S2).

Levels of γ-globin mRNA did not change either after pre-miR overexpression or following anti-miR overexpression; only shRNA overexpression led to 50% reduction of γ-globin mRNA levels (Figure S3 C&D).

### miR-96 targets the γ-globin ORF

**Figure 5:**
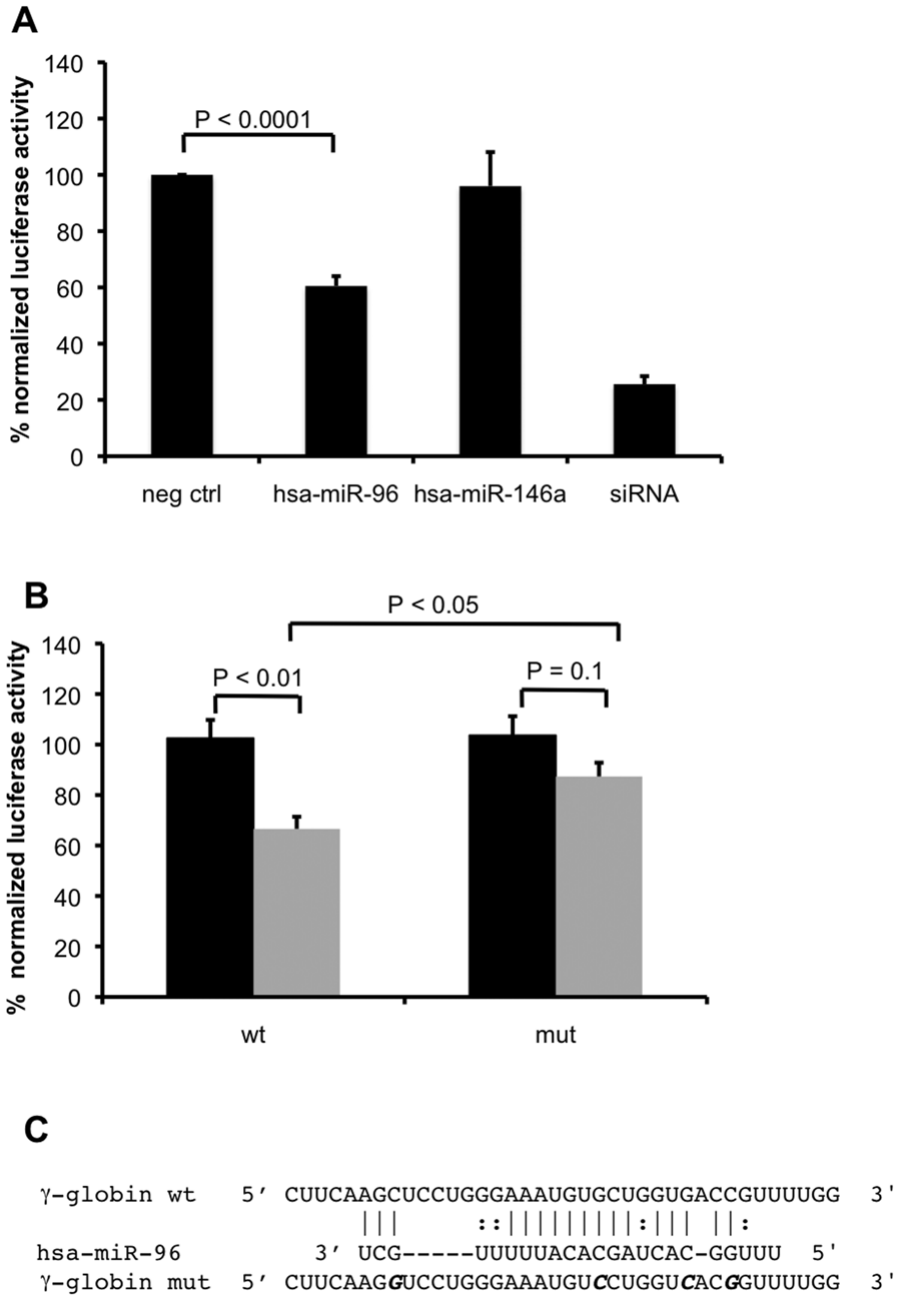
miR-96 targets the γ-globin ORF. HEK239T cells were co-transfected with the psiCHECK-2 reporter vector expressing Renilla luciferase fused to γ-globin cDNA and the control mimic (neg ctrl), miR-96 mimic, miR-146a mimic or small interfering RNA against γ-globin (siRNA). (A) Relative Renilla luciferase activity in cell lysates normalized to firefly luciferase are shown. (B) Relative Renilla luciferase activities after co-transfection of the control mimic (*black*) or miR-96 mimic (*grey*) with wild-type (*wt*) γ-globin cDNA or mutated γ-globin cDNA (*mut*) at the predicted miR-96 target site are represented. Values represent mean±SEM of six experiments. P values were determined by the Student's t-test. (C) Predicted miR-96 binding site within the ORF of γ-globin and binding site mutations tested (indicated in bold italics).

Finally, we performed luciferase reporter assays to investigate whether miR-96 directly interacts with γ-globin mRNA (Figure 3, 4). Therefore, a vector was constructed harboring the γ-globin cDNA downstream of the *Renilla* luciferase ORF (psiCHECK-2-γ-globin). A second reporter gene, firefly luciferase, present in psiCHECK-2 allowed the normalization of *Renilla* luciferase activity. Human embryonic kidney (HEK) cells were co-transfected with the reporter construct, and with pre-miRs for either miR-96 or miR-146a. For miR-96, but not for miR-146a, a significant decrease in the relative levels of Renilla luciferase activity compared to those of Firefly luciferase activity was measured (Figure 5A). In addition, the disruption of the predicted miR-96 target site within the γ-globin coding sequence revealed luciferase activity levels equal to those of the negative control samples, indicating that miR-96 directly and sequence specifically targets the ORF of γ-globin mRNA at the predicted target site (Figure 5B&C).

## Discussion

In the present study we identified miR-96 as a direct inhibitor of γ-globin expression. Initially, we found that in CB reticulocytes, the amount of γ-globin mRNA bound by AGO2 miRISC was 15 times less compared to AGO2 miRISC from AB reticulocytes. On the basis of these results, we speculate that miRISC binds residual γ-globin mRNAs that are still transcribed during erythropoiesis in adult after the globin switch has been completed, thus leading to an inhibition of γ-globin expression.

Since γ-globin mRNA is associated with miRISC in AB reticulocytes and therefore likely interacts with miRNAs, we investigated miRNA expression patterns in CB and AB. We were able to identify miR-96, miR-146a, let-7a, 330-3p and miR-888 as being present in significantly higher amounts in AB reticulocytes compared to those from CB and therefore as being potential inhibitors of γ-globin expression. Consistently, a previous report on miRNA expression profiling in CB and AB also identified miR-96 and let-7a as being significantly less expressed in reticulocytes from CB [18]. However only miR-96 was enriched significantly more in immunoprecipitated AGO2 complexes from adult reticulocytes compared to cord blood; let-7a did not show this pattern, indicating that it might be less probable, that let-7a directly targets γ-globin mRNAs.

In addition we were able to elucidate the role of these miRNAs for the HbF expression in further detail. In primary erythroid cultures, only overexpression of miR-96 led to a 50% decrease of γ-globin protein expression in CB-derived erythropoietic cells expressing high amounts of γ-globin protein. Even more important, the knockdown of the physiological miR-96 levels in BM-derived erythropoietic cells, expressing low amounts of γ-globin mRNA and thus low amounts of HbF, led to a significant increase of 20% in γ-globin protein expression. Neither overexpression nor knock down of the other miRNAs led to a change of γ-globin expression.

Further experiments then allowed us to demonstrate that miR-96 in complex with AGO2 binds to a seedless but highly complementary target site within the open reading frame of γ-globin mRNA. Although most miRNAs are believed to target mRNAs in their 3′-UTRs, both seedless target sites and target sites located within the ORF have been previously reported [34], [35], [36], [37], [38].

To date, miR-96 has been shown to be crucial for the development of the inner ear and hearing [39], [40], and to inhibit the expression of the platelet protein VAMP8 [41]. In addition up-regulation of miR-96 has been associated with the transformation or maintenance of breast cancer cells through the expression inhibition of the transcription factor FOXO1 [42].

During the postnatal globin switch, chromatin remodeling of the β-globin locus leads to an almost complete change from γ-globin transcription to β-globin transcription. A number of transcription factors such as GATA-1 [43], EKLF [44], [45], cMYB [46], or BCL11A [47] have been described as being required for the transcriptional switch from γ-globin to β-globin expression. Further, c-kit ligand activity has been shown to play a role in the HbF switching: a recent study from Gabbianelli et al. [20] reported a gradual increase in miR-221 and miR-222 expression from pre-term to full-term CB to adult hematopoietic progenitor cells which down-modulates c-kit. In support of this report we identified miR-222 to be significantly 20 times less abundant in reticulocytes from CB compared to AB (Figure 3).

However in adult erythropoiesis after the switch some residual amounts of γ-globin mRNAs are still transcribed and can be detected in reticulocytes [48], [49]. Here we demonstrate that these few copies of γ-globin mRNA in AB reticulocytes, about 40 times less than in CB, are targeted by miR-96 leading to a further repression of HbF expression.

Our study seems to be limited by the relative small changes of γ-globin expression after knock-down of endogenous miRNAs. However, the transduction with anti-miRs led only to a decrease of 50% of endogenous miR-96. Therefore we speculate that the residual endogenous miR-96 most likely continues to inhibit γ-globin expression. In addition, a study on the role of miRNAs for the erythropoietic enucleation showed also changes of 15–25% after miRNA depletion [50]. Relatively small but significant changes can be expected by manipulating single miRNAs, as not yet identified miRNAs might also target the γ-globin mRNA. Thus, the concerted knock-down of several miRNAs could lead to a more pronounced increase of γ-globin expression. To investigate effects of microRNAs in complex with AGO2 on the globin expression, AGO2 knock downs in erythropoietic cells could have been investigated. However we refrained from knocking down AGO2 as it was well documented that AGO2 is essential for a normal erythropoiesis [51] Nonetheless, our results suggest that translation of residual γ-globin mRNA in adult erythropoiesis is repressed by miR-96 in complex with AGO2, adding a fine-tuning mechanism of globin gene regulation.

Our finding that expression of HbF is repressed by miR-96 raises intriguing questions as to what extent this regulation contributes to the phenotypic heterogeneity observed in β-thalassemia and sickle cell disease. Currently, we are studying the possibility that altered miRNA expression, including that of miR-96, might contribute to different HbF levels observed among patients. Along these lines, our preliminary data on RBCs from a small cohort of sickle cell patients (Azzouzi et al. unpublished data) indicate that miR-96 expression levels inversely correlate with HbF content,. Moreover, a recent study with sickle cell disease patients after butyrate treatment showed increased binding of γ-globin mRNA to ribosomes in reticulocytes [7]; one could speculate that this drug might interfere with the binding of miRNAs and AGO proteins to γ-globin mRNA, leading to increased HbF expression.

In conclusion, we have been able to demonstrate direct regulation of γ-globin expression by miR-96. Further studies are needed to assess if miRNA regulation also interferes with the hemoglobin switch occurring during pre- and postnatal development. In addition, the possibility of using miRNA inhibitors for the therapeutic induction of γ-globin in patients with hemoglobinopathies needs to be studied.

## Materials and Methods

### Blood sampling and reticulocyte purification

The institutional ethics board of the University Children's Hospital, Zurich and of the Canton of Zurich approved the study protocol, and all subjects provided written informed consent to participate in accordance with the Declaration of Helsinki. The venous blood samples were collected during routine blood tests. For the venipuncture, a cream composed of 5% lidocaine and 5% prilocaine (EMLA; Astra Zug, Switzerland) was applied. Five to 10 ml of venous blood was collected into heparin. The blood was washed three times with 10 ml of phosphate-buffered saline containing 2 mM Ethylenediaminetetraacetic acid (EDTA) and separated in a Ficoll-Hypaque gradient (GE Healthcare, Glattbrug, Switzerland) to remove mononuclear cells and platelets. Reticulocytes were then filtered through a leuko-depletion filter (Purecell Neo; Pall, Basel, Switzerland) (Figure S1 A). The leukodepleted reticulocytes were washed and resuspended in phosphate-buffered saline/EDTA. The purity grade of reticulocytes was assessed by an automated blood cell analyzer (Sysmex Digitana, Horgen, Switzerland), as well as by flow cytometry for surface expression of CD45 with fluorescence-labeled antibodies (Becton Dickinson, Rotkreuz, Switzerland) (Figure S1 B). In addition, depletion of leukocytes and platelets, and enrichment of reticulocytes was analyzed by quantitative real time PCR (qPCR) of the pan-leukocyte marker CD45 as well as of mRNAs encoding different hemoglobin subunits (Figure S1 C).

### Cell cultures

Erythroid cells were cultured using a 2-phase liquid system. Mononuclear cells isolated from umbilical cord blood and bone marrow (Stemcell Technologies, Grenoble, France) and cultured for 7 days in phase I medium consisting of serum-free StemSpan (Stem Cell Technologies, Vancouver, BC) supplemented with 100 ng/ml fetal liver tyrosine kinase 3 ligand, 100 ng/ml thrombopoietin and 100 ng/ml stem cell factor (ProSpec, Rehovot, Israel). Cells were incubated at 37°C, 5% CO_2_. After 7 days, nonadherent cells were collected and reseeded at a concentration of 5×10^5^ cells/ml in phase II medium (StemSpan supplemented with 50 ng/ml insulin-like growth factor-1, 50 ng/mL stem cell factor, and 3 U/ml human recombinant erythropoietin (Merck, Darmstadt, Germany). Cell samples were collected from phase II cultures after 8, 11 and 14 days of culture.

K562 and HEK293T were grown in IMDM (PAA, Coelbe, Germany) supplemented with 10% fetal bovine serum, 4 mM glutamine, and 1× Antibiotic-Antimycotic reagent (Invitrogen AG, Basel, Switzerland). HbF induction in K562 cells was performed, as described [27], [28], [29] with minor modifications, by adding 50 µM of hemin (Sigma, Buchs, Switzerland) to the medium for two days.

### AGO co-immunoprecipitation

Co-immunoprecipitation experiments were performed as described [25], [52] with modifications: 500 µl of packed RBCs were lysed with 1.5 ml of lysis buffer (20 mM Tris-HCl pH 7.5, 150 mM NaCl, 0.5% IGEPAL, 2 mM EDTA, 0.5 mM DTT, heparin 0.2 mg/ml, one tablet protease inhibitor (Roche, Rotkreuz, Switzerland), 50 U/ml RNase OUT™ (Invitrogen AG, Basel, Switzerland), 50 U/ml Superase•IN™ (Applied Biosystems, Rotkreuz, Switzerland)). Lysates were cleared by centrifugation at 14,000 *g* for 10 minutes three times. Twenty mg of cell extract protein were used for each experiment. AGO2 monoclonal antibody-containing hybridoma (11A9) supernatant (2.5 ml) was coupled to 50 µl protein G-Sepharose (GE Healthcare, Switzerland) overnight at 4°C. Coupled beads were washed three times with NT2 buffer (50 mM Tris-HCl pH 7.5, 300 mM NaCl, 5 mM MgCl_2_, 0.05% IGEPAL) and resuspended in NT2-RIP (NT2 buffer supplemented with 12.5 µl RNase OUT™ (50 U/ml), 25 µl Superase•IN™ (50 U/ml), 20 µl 1 M DTT (2 mM), 4 µl heparin 0.02 mg/ml). Beads were then incubated with RBC lysates for six hours at 4°C. IP samples were washed three times with NT2 buffer and proteins were eluted with SDS-EDTA solution (50 mM Tris pH 8, 100 mM NaCl, 10 mM EDTA, 1% SDS) at 65°C for 15 minutes. An aliquot of each eluate was kept for immunoblotting and the remainder was used for RNA isolation using the mirVana™ microRNA isolation kit (Applied Biosystems, Rotkreuz, Switzerland).

Following electrophoresis by SDS-PAGE the gel slices corresponding to the size of AGO2 (100 kDa) were cut out and dehydrated in 100% acetonitrile. Proteins were in-gel digested using Sequencing Grade trypsin (Promega, Dübendorf, Switzerland) as described by Shevchenko [53]. The peptide samples were then analyzed on an Agilent 1100 micro HPLC system (Agilent, Morges, Switzerland) coupled to an LTQ linear ion trap mass spectrometer (Thermo Electron, San Jose, CA) equipped with a nanoelectrospray ion source (Thermo Electron, San Jose, CA). Peptides were separated on an RP-HPLC column (10 cm length and 75 µm inner diameter) packed with C18 resin (Magic C18 AQ 3 µm; Michrom Bioresources) with a linear gradient from 95% buffer A (water, 0.1% formic acid) and 5% buffer B (water, 0.1% formic acid and 90% Acetonitrile) to 60% buffer A and 40% buffer B at a flow rate of 0.5 µL/min. The data acquisition mode was set to acquire one MS scan followed by three collision induced dissociation MS/MS scans. The MS full scans were recorded over a mass range of 400–1600 m/z. The normalized collision energy was set to 35%.

Acquired raw data files were converted with ReAdW into mzXML files [54] which were searched with Sorcerer-SEQUEST [55] against the human protein database of the UniProtKB/Swiss-Prot Protein Knowledgebase (Version 57.15). The Trans-Proteomic Pipeline TPP [56] v4.0 JETSTREAM rev 2 including PeptideProphet [57] and ProteinProphet [58] was used for the statistical analysis of the search results. The false discovery rate was set to 1%, corresponding to a ProteinProphet probability score of 0.9. The remaining set of proteins was evaluated manually and common contaminations were eliminated.

### Quantitation of mRNAs and miRNAs

α-, β- δ- and γ-globin mRNAs, as well as control GAPDH mRNA, were quantified using the TaqMan® Gene Expression Cells-to-CT™ Kit and specific primers (Applied Biosystems, Rotkreuz, Switzerland). To determine the mRNA copy numbers of the target gene, Ct-values were measured for serial dilutions (300,000, 30,000, 3,000, 300 and 30 copies) of globin cDNA or GAPDH cDNA. Using the equation of the standard curves for each gene the actual copy number of globin genes and GAPDH were calculated for each sample. The globin data were normalized by calculating the ratio between globins and GAPDH.

For miRNA expression profiling, total RNA samples were prepared from RBCs and K562 cells using the mirVana™ microRNA isolation kit (Applied Biosystems, Rotkreuz, Switzerland) following the manufacturer's protocol. For each sample, 360 ng of total RNA were reverse transcribed using the Megaplex™ Pools (Applied Biosystems, Rotkreuz, Switzerland) and screened for the presence of 350 known human miRNAs by qPCR using low-density arrays (Applied Biosystems, Rotkreuz, Switzerland). For the resulting Ct values for each miRNA, a complementary Ct^c^ (40–Ct) was calculated; the normalized ΔCt^c^ (Ct^c^_gene_–Ct^c^_endogenous control_) value heatmaps were then generated.

For single miRNA analysis, 25 ng of RNA isolated from cultured erythroid cells were used for real time PCR quantification using the TaqMan® MicroRNA Cells-to-CT™ Kit and specific primers (Applied Biosystems, Rotkreuz, Switzerland) according to the manufacturer's instructions.

### Immunoblotting and ELISA

RBC lysates in protein extraction buffer (4 mM CaCl_2_, 4 mM MgCl_2_, 1% Triton, 20 mM HEPES, 2 mM PMSF) or eluates from AGO co-immunoprecipitation were used for immunoblotting. Protein samples were resolved on NuPAGE 4–12% Bis-Tris polyacrylamide gels (Invitrogen AG, Basel, Switzerland), transferred to PVDF membrane and blocked with 5% non-fat dried milk in Tris-buffered saline (20 mM Tris, 150 mM NaCl, pH 7.4) with 0.1% Tween-20. Membranes were incubated with the following primary antibodies: rat anti-human AGO1, AGO2, AGO3, AGO4 [12], [24], [26], β-globin, γ-globin, β-actin (Santa Cruz Biotechnology, Heidelberg, Germany), followed by HRP-conjugated secondary antibodies (Jackson Immunology, Newmarket, UK) and developed with ECL reagent (GE Healthcare, Switzerland).

γ-globin ELISA was performed using cell lysates in ELISA lysis buffer (50 mM Tris-HCl pH 7.5, 150 mM NaCl, 0.5% IGEPAL) with the Human Fetal Hemoglobin ELISA Quantification kit (Bethyl, Montgomery, TX, USA) according to the manufacturer's protocol.

### miRNA overexpression and miRNA knockdown

Pseudoviral particles were generated using lentivector-based miRNA precursor and miRZIP™ lentivector-based anti-miRNA constructs following the manufacturer's instructions (System Biosciences, Mountain View, CA). Retroviral particles were prepared from a mixture of 4 HuSH 29mer shRNA constructs against the γ-globin mRNA (Origene Technologies, Inc., Rockville, MD, USA) and used as control. To achieve stable expression during erythropoiesis primary erythroid cultures were transduced with pseudoviral particles (10/cell) containing vectors coding for miRNA precursors or anti-miRNAs after 8 days of culture. A transduction efficiency of 90% was achieved.

### Luciferase reporter assay

The γ-globin cDNA was subcloned from the TrueClone NM_000559.2 vector (Origene, Rockville, MD, USA) into the psiCHECK-2 vector (Promega AG, Duebendorf, Switzerland). The γ-globin mir-96 mutant reporter was constructed with Phusion™ Site-Directed Mutagenesis (Finnzymes, Espoo, Finland), which created a four base pair change in the mir-96 target site (bold and underlined) (AAGCUCCUGGGAAAUGUGCUGGUGACCGU to replace AAGGUCCUGGGAAAUGUCCUGGUCACGGU).

To evaluate the effect of miR-96 and miR-146a on γ-globin activity, we used precursor miRNA (Applied Biosystems, Rotkreuz, Switzerland). HEK293T cells were co-transfected with 10 ng psiCHECK reporter and 500 nM of miRNA precursors for each experiment, using siPORT NeoFX Transfection Agent (Applied Biosystems, Rotkreuz, Switzerland). After 24 hours, the transfected cells were washed and lysed with passive lysis buffer (Promega AG, Duebendorf, Switzerland). The luciferase activities (firefly and Renilla) were then determined by a luminometer (Berthold Technology, Regensdorf, Switzerland) using the Dual luciferase reporter assay kit (Promega AG, Duebendorf, Switzerland). The relative reporter activities were calculated by normalization of firefly to the Renilla luciferase activities determined in the same lysates. To confirm the target site of miR-96 on the γ-globin cDNA, its target site was cloned into the psiCHECK-2 vector as well as the mutated target site.

## Supporting Information

Figure S1 **Purification of reticulocyte.** (A) The venous blood or umbilical cord blood samples were collected into heparin and leukodepleted by Ficoll density gradient and filtration. (B) The purity of reticulocytes was assessed by flow cytometry and (C) by real-time PCR. (B) Following leukodepletion, cells were stained with an antibody against CD45, which is specific to leukocytes. No CD45-positive cells were detected after leukodepletion by flow cytometry. (C) The analysis of purified reticulocytes at the mRNA level showed no CD45 mRNA after leukodepletion, whereas all globin mRNAs were detected. All subsequent experiments were perform with reticulocytes containing both reticulocytes and mature erythrocytes, in order not to lose any reticulocytic RNA from the relatively small blood samples. HBA, α-globin; HBB, β-globin; HBD, δ-globin; HBG, γ-globin; PBMC, peripheral blood mononuclear cells; reti, leukodepleted reticulocytes; WB, whole blood.
(TIF)

Figure S2 **Analysis of immunopurified AGO2 by LC-MS/MS.** (A) Amino acid sequence of human AGO2. Tryptic peptides identified by LC-MS/MS are highlighted. (B) Representative MS/MS spectrum, amino acid sequence and annotated fragment ions from an identified human AGO2 peptide.
(TIF)

Figure S3 **RNA and protein levels in erythroid cell cultures after overexpression and knockdown of miRNAs.** (A) Relative quantification of miRNAs after transduction of miRNA-precursors and (B) after transduction of anti-miRNAs. miRNA levels in erythropoietic cells transduced with negative control were given a relative value of 1.0. All levels of overexpressed miRNAs were expressed as n-fold change compared with the negative control. (C) Quantification of γ-globin mRNA after transduction of miRNA-precursors and (D) after transduction of anti-miRNAs. All γ-globin mRNA quantities were expressed as copy numbers per cell. Cells were harvested and analyzed at day 8 (*d8*), 11 (*d11*) and 14 (*d14*). Values represent three independent experiments (n = 3). (E) Western blot analysis of γ-globin and β-globin in CB-derived erythroid cultures transduced with miR-96 precursors (*miR-96*) and BM-derived erythroid cultures transduced with anti-miR-96. As negative control (*neg ctrl*), cells were transduced with empty vector. Actin was included as loading control.
(TIF)

Table S1 Ct values obtained by real time PCR.
(XLS)

Table S2 Quantification of morphological development stages of erythropoietic cells.
(PDF)
