# Supplementary figures and images for: MicroRNA-96 Directly Inhibits γ-Globin Expression in Human Erythropoiesis
Source: PLoS One. 2011 Jul 28;6(7):e22838. doi: 10.1371/journal.pone.0022838 (PMC3145767; doi:10.1371/journal.pone.0022838)

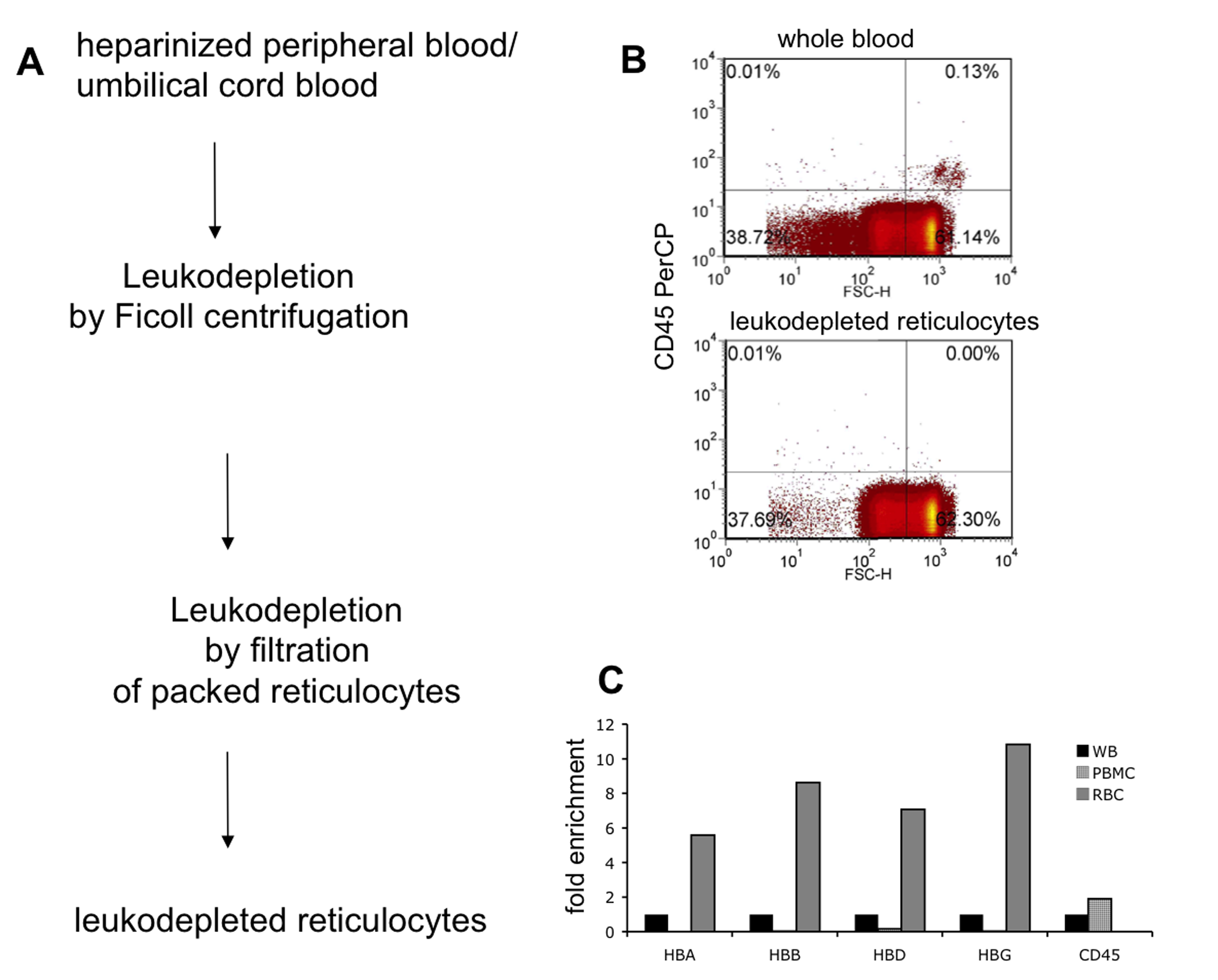

Supplement: Figure S1 — Purification of reticulocyte. (A) The venous blood or umbilical cord blood samples were collected into heparin and leukodepleted by Ficoll density gradient and filtration. (B) The purity of reticulocytes was assessed by flow cytometry and (C) by real-time PCR. (B) Following leukodepletion, cells were stained with an antibody against CD45, which is specific to leukocytes. No CD45-positive cells were detected after leukodepletion by flow cytometry. (C) The analysis of purified reticulocytes at the mRNA level showed no CD45 mRNA after leukodepletion, whereas all globin mRNAs were detected. All subsequent experiments were perform with reticulocytes containing both reticulocytes and mature erythrocytes, in order not to lose any reticulocytic RNA from the relatively small blood samples. HBA, α-globin; HBB, β-globin; HBD, δ-globin; HBG, γ-globin; PBMC, peripheral blood mononuclear cells; reti, leukodepleted reticulocytes; WB, whole blood. (TIF) [file pone.0022838.s001.tif]

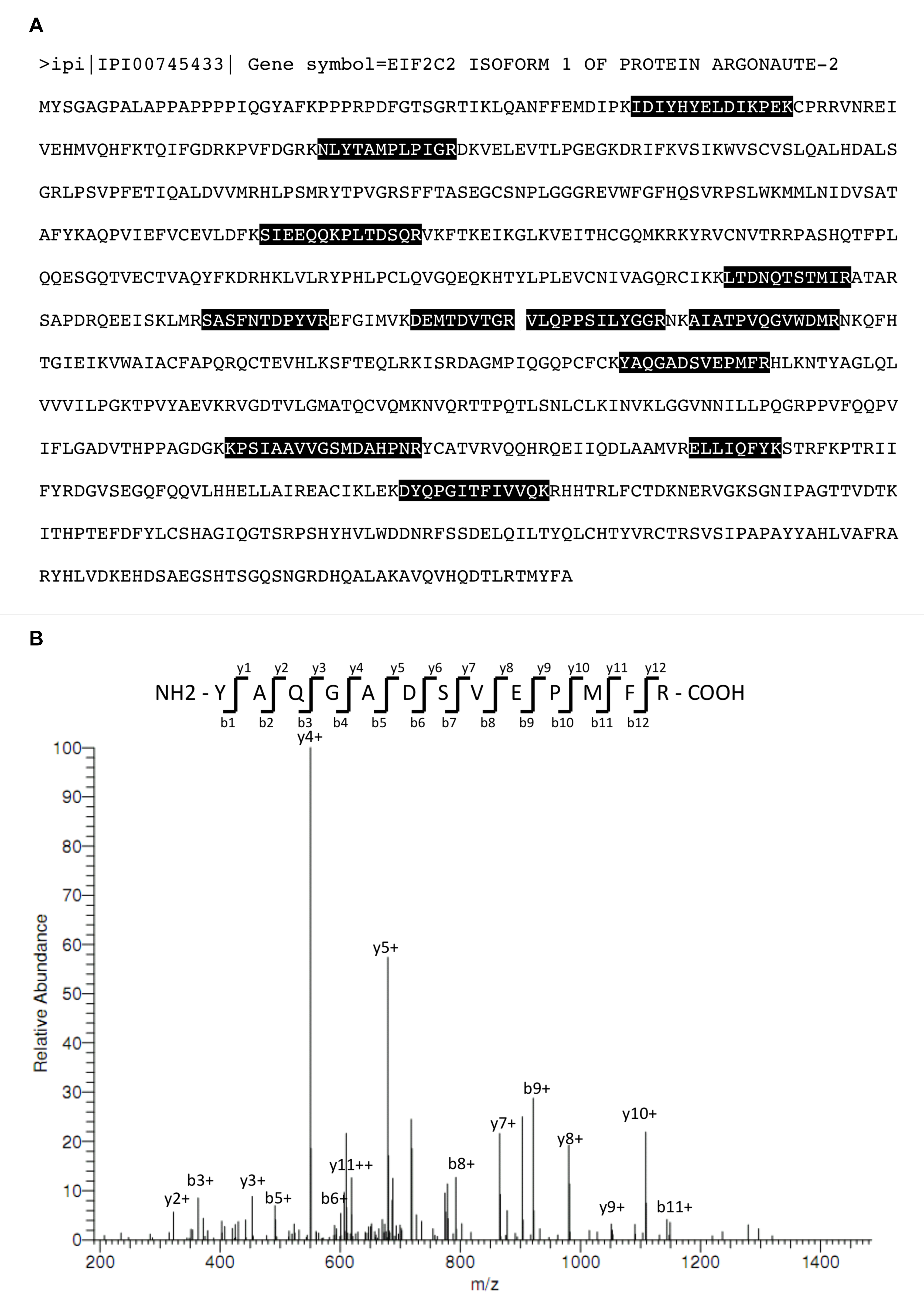

Supplement: Figure S2 — Analysis of immunopurified AGO2 by LC-MS/MS. (A) Amino acid sequence of human AGO2. Tryptic peptides identified by LC-MS/MS are highlighted. (B) Representative MS/MS spectrum, amino acid sequence and annotated fragment ions from an identified human AGO2 peptide. (TIF) [file pone.0022838.s002.tif]

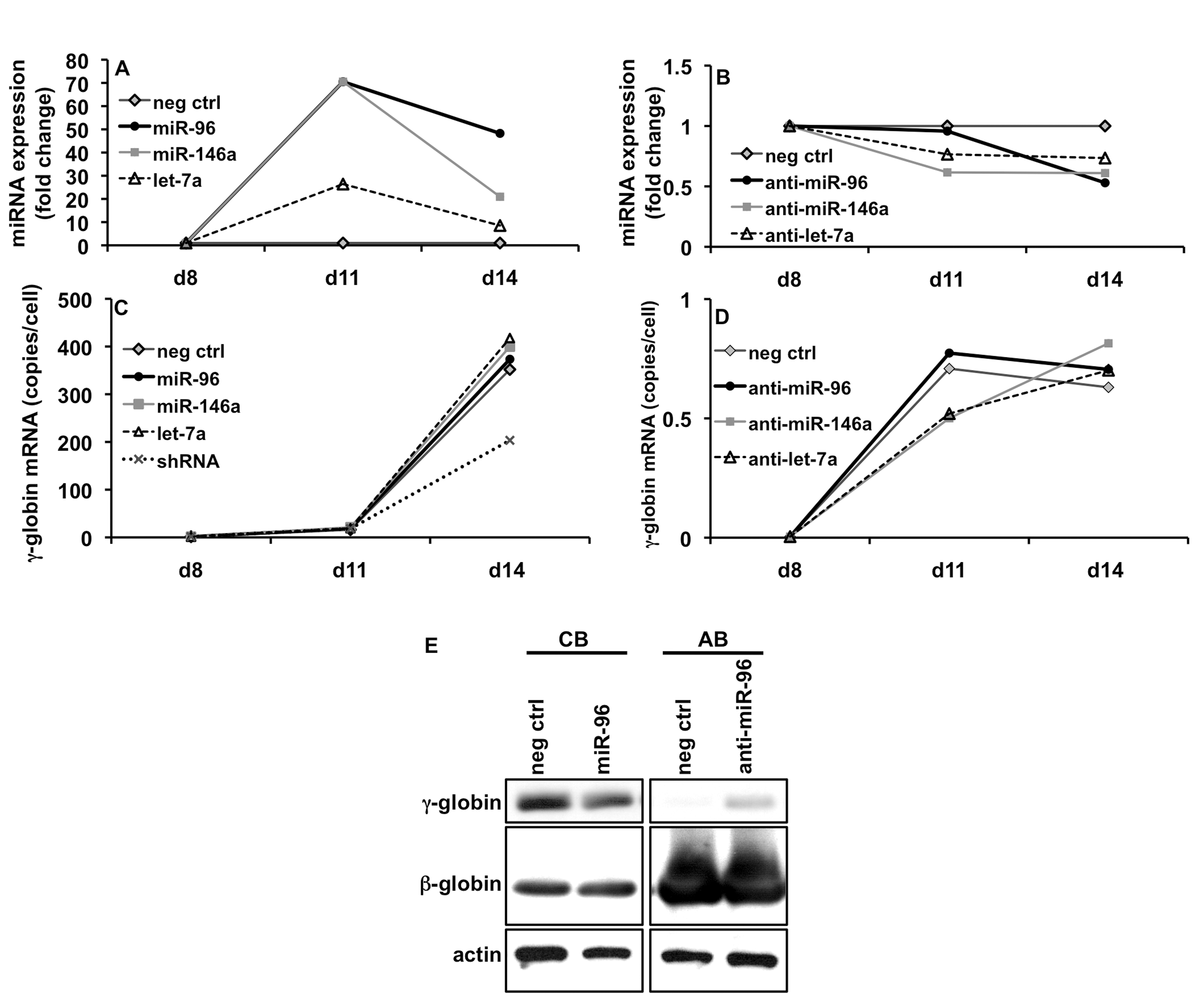

Supplement: Figure S3 — RNA and protein levels in erythroid cell cultures after overexpression and knockdown of miRNAs. (A) Relative quantification of miRNAs after transduction of miRNA-precursors and (B) after transduction of anti-miRNAs. miRNA levels in erythropoietic cells transduced with negative control were given a relative value of 1.0. All levels of overexpressed miRNAs were expressed as n-fold change compared with the negative control. (C) Quantification of γ-globin mRNA after transduction of miRNA-precursors and (D) after transduction of anti-miRNAs. All γ-globin mRNA quantities were expressed as copy numbers per cell. Cells were harvested and analyzed at day 8 (d8), 11 (d11) and 14 (d14). Values represent three independent experiments (n = 3). (E) Western blot analysis of γ-globin and β-globin in CB-derived erythroid cultures transduced with miR-96 precursors (miR-96) and BM-derived erythroid cultures transduced with anti-miR-96. As negative control (neg ctrl), cells were transduced with empty vector. Actin was included as loading control. (TIF) [file pone.0022838.s003.tif]
