# Supplementary material for: MicroRNA-96 Directly Inhibits γ-Globin Expression in Human Erythropoiesis
Source: PLoS One. 2011 Jul 28;6(7):e22838. doi: 10.1371/journal.pone.0022838 (PMC3145767; doi:10.1371/journal.pone.0022838)
Supplement: Table S2 — Quantification of morphological development stages of erythropoietic cells. (PDF) [file pone.0022838.s005.pdf]

Table S2 Quantification of developmental stages of in vitro derived erythropoietic cells.
For each condition 200 cells were counted (n=4) and different differentiation stages were expressed as % of the total number of counted cells.

CB

pre-miR vector	erythropoietic cells	days in culture: 8	11		14	
neg ctrl	myelocytic cell	29.3 ± 8.7	0.7 ± 1.2	p>0.05	0.1 ± 0.2	p>0.05
	Proerythroblast	41.5 ± 4.6	20.2 ± 3.0	p>0.05	0.9 ± 1.6	p>0.05
	Early erythroblast	28.2 ± 12.1	78.0 ± 3.9	p>0.05	43.6 ± 1.7	p>0.05
	intermediate erythroblast	0.9 ± 0.9	1.2 ± 1.3	p>0.05	30.6 ± 1.4	p>0.05
	late erythroblast	0.0 ± 0.0	0.0 ± 0.0	p>0.05	24.7 ± 2.3	p>0.05
miR-96	myelocytic cell	n.a.	0.0 ± 0.0	p>0.05	0.4 ± 0.7	p>0.05
	Proerythroblast	n.a.	14.6 ± 7.9	p>0.05	2.2 ± 3.9	p>0.05
	Early erythroblast	n.a.	82.4 ± 6.2	p>0.05	42.8 ± 4.0	p>0.05
	intermediate erythroblast	n.a.	2.5 ± 2.2	p>0.05	30.5 ± 5.7	p>0.05
	late erythroblast	n.a.	0.5 ± 0.4	p>0.05	24.1 ± 2.3	p>0.05
miR-146a	myelocytic cell	n.a.	0.7 ± 1.1	p>0.05	0.3 ± 0.5	p>0.05
	Proerythroblast	n.a.	23.1 ± 4.7	p>0.05	1.3 ± 1.5	p>0.05
	Early erythroblast	n.a.	72.6 ± 8.8	p>0.05	33.8 ± 12.4	p>0.05
	intermediate erythroblast	n.a.	3.1 ± 4.6	p>0.05	50.3 ± 6.0	p>0.05
	late erythroblast	n.a.	0.5 ± 0.9	p>0.05	14.2 ± 5.5	p<0.05
let-7a	myelocytic cell	n.a.	2.6 ± 2.8	p>0.05	0.2 ± 0.3	p>0.05
	Proerythroblast	n.a.	27.5 ± 3.7	p<0.05	0.5 ± 0.8	p>0.05
	Early erythroblast	n.a.	67.7 ± 2.5	p<0.05	55.7 ± 6.2	p<0.05
	intermediate erythroblast	n.a.	2.2 ± 2.3	p>0.05	36.7 ± 6.7	p>0.05
	late erythroblast	n.a.	0.0 ± 0.0	p>0.05	7.1 ± 1.2	p<0.001

BM

anti-miR vector	erythropoietic cells	days in culture: 8	11		14	
neg ctrl	myelocytic cell	31.4 ± 0.6	3.6 ± 1.3	p>0.05	0.6 ± 1.1	p>0.05
	Proerythroblast	41.9 ± 6.2	29.1 ± 2.0	p>0.05	0.0 ± 0.0	p>0.05
	Early erythroblast	26.3 ± 7.1	67.4 ± 0.8	p>0.05	50.2 ± 2.0	p>0.05
	intermediate erythroblast	0.4 ± 0.7	0.0 ± 0.0	p>0.05	37.0 ± 1.4	p>0.05
	late erythroblast	0.0 ± 0.0	0.0 ± 0.0	p>0.05	12.2 ± 1.3	p>0.05
anti-miR-96	myelocytic cell	n.a.	3.9 ± 1.6	p>0.05	0.3 ± 0.5	p>0.05
	Proerythroblast	n.a.	31.2 ± 8.4	p>0.05	0.5 ± 0.9	p>0.05
	Early erythroblast	n.a.	64.0 ± 5.6	p>0.05	45.2 ± 2.2	p>0.05
	intermediate erythroblast	n.a.	1.0 ± 1.7	p>0.05	42.5 ± 2.7	p>0.05
	late erythroblast	n.a.	0.0 ± 0.0	p>0.05	11.5 ± 1.3	p>0.05
anti-miR-146a	myelocytic cell	n.a.	5.4 ± 3.5	p>0.05	0.1 ± 0.2	p>0.05
	Proerythroblast	n.a.	28.3 ± 3.0	p>0.05	1.6 ± 1.0	p>0.05
	Early erythroblast	n.a.	64.3 ± 2.6	p>0.05	39.3 ± 3.1	p>0.05
	intermediate erythroblast	n.a.	2.0 ± 3.5	p>0.05	41.6 ± 8.6	p>0.05
	late erythroblast	n.a.	0.0 ± 0.0	p>0.05	17.4 ± 4.3	p>0.05
anti-let-7a	myelocytic cell	n.a.	2.8 ± 4.4	p>0.05	0.0 ± 0.0	p>0.05
	Proerythroblast	n.a.	26.5 ± 5.6	p>0.05	0.2 ± 0.3	p>0.05
	Early erythroblast	n.a.	69.3 ± 9.3	p>0.05	32.3 ± 6.7	p<0.05
	intermediate erythroblast	n.a.	1.3 ± 2.3	p>0.05	47.8 ± 5.4	p<0.05
	late erythroblast	n.a.	0.0 ± 0.0	p>0.05	19.8 ± 2.8	p<0.05

n.a.: not applicable
neg ctrl: negative control transduced with an empty vector
p- values: student t-test compared to neg ctrl
